# Impact of clomazone on bacterial communities in two soils

**DOI:** 10.3389/fmicb.2023.1198808

**Published:** 2023-07-31

**Authors:** Hairong He, Jiarui Huang, Zhenzhu Zhao, Weisheng Feng, Xiaoke Zheng, Pengqiang Du

**Affiliations:** ^1^College of Pharmacy, Henan University of Chinese Medicine, Zhengzhou, China; ^2^College of Plant Protection, Henan Agricultural University, Zhengzhou, China

**Keywords:** clomazone, bacterial network, network composition, stability, dissimilarity

## Abstract

**Introduction:**

Bacterial communities are important for soil functions, but the effect of clomazone on network complexity, composition, and stability is not well studied.

**Method:**

In this study, two agricultural soils were used to test the impact of clomazone on bacterial communities, and the two soils were treated with three concentrations of clomazone (0, 0.8, 8, and 80 mg kg^1^) in an incubator.

**Results and discussion:**

Bacterial network nodes, links, and average degrees were all decreased by 9–384, 648–829, and 0.703–2.429, respectively. Based on keystone nodes, the topological roles of the nodes were also influenced by clomazone. Bacterial network composition was also impacted based on the analysis of similarity (ANOSIM) and network dissimilarity. Compared with control and clomazone treatments in both soils, the ANOSIM between control and all clomazone treatments was higher than 0.6, network dissimilarities were 0.97–0.98, shared nodes were 131–260, and shared links were 12–100. The bacterial network stability was decreased by clomazone, with decreased robustness by 0.01–0.016 and increased vulnerability by 0.00023–0.00147 in both soils. There were fewer bacterial network modules preserved after clomazone treatment, and the bacterial network community functions were also impacted in both soils. Based on these results, soil bacterial species connections, modularization, and network stability were significantly impacted by clomazone.

## 1. Introduction

The soil microorganismal community is an interconnected unity through various and complicated relationships, such as mutualism, commensalism, parasitism, neutral predation, competition, and amensalism (Faust and Raes, 2012; Coyte et al., 2015). For all these processes, soil microorganisms perform functions by maintaining material, energy, and information exchange (Montoya et al., 2006; Glaze et al., 2022) and are fundamental to organic-matter degradation, pollution control, agricultural production, groundwater quality keeping, nitrogen cycling, and greenhouse gas regulation (Falkowski et al., 2008; Li et al., 2021). Therefore, intermicrobial connections are vital for maintaining homeostasis in soil processes. Network analysis has been used to characterize the complex ecological relationships among microbial species, and network nodes and links are used to represent species and their relationships, respectively (Przulj and Malod-Dognin, 2016). Therefore, networks are useful for examining species relationships and ecosystem processes (Berry and Widder, 2014).

Soil microbial community networks are threatened by many challenges such as global warming (Yuan et al., 2021; Zhu et al., 2022), soil erosion (Qiu et al., 2021), and terrestrial pollution (Du et al., 2021). Network composition is important for the stability of soil processes. Among the factors that may perturb the integrity of soil microbial networks, pesticide application has become a substantial threat yet is a standard practice in modern agriculture. Indeed, previous studies have shown that pesticides have a direct impact on microbial communities and their functions (Lerner et al., 2020; Li et al., 2020; Liu et al., 2020; Qiao et al., 2020; Yang, 2021). For example, researchers have analyzed the effect of pesticides on soil microbial network topological indices (Gao et al., 2018). However, studies related to node persistence, microbial network composition, and stability were limited. It is well known that soil microbial composition is the base of soil ecology function. Therefore, the analysis of changes in network composition in response to pesticides has important implications on soil community functions.

Clomazone {2-[(2-chlorophenyl) methyl]-4,4-dimethyl-1,2-oxazolidin-3-one} is an isoxazolidinone compound commonly used as a selective herbicide for many crops, and it has a half-life of >195 days in the field (PPDB^1^). Previous reports showed that clomazone can influence soil microbial communities (Du et al., 2018), indicating that the network structure can be altered, yet no study has been carried out concerning whether clomazone can affect microbial networks. To address this issue, we carried out a microcosmic experiment indoors over a period of 3 months. In this study, network complexity, dissimilarity, network stability, and preserved modules were used to evaluate the impact of clomazone on bacterial network composition and stability. In addition, the correlations between functions and the network community were also analyzed to ascertain whether the functions were changed. These indices will reflect the impact of clomazone on bacterial network.

## 2. Materials and methods

### 2.1. Experimental design

There were two soils from the Jiansanjiang reclamation area (JSJ) and the Langfang research base of the Chinese Academy of Agricultural Sciences (LF). According to soil particle diameter, the soil form JSJ was identified as silty clay, and the soil form LF was identified as silty loam. The silty loam had 18 g organic matter kg^−1^, 74.9 mg available P kg^−1^, 289.8 mg available K kg^−1^, and a pH of 7.07; the silty clay had 25.8 g organic matter kg^−1^, 51.7 mg available P kg^−1^, 289.8 mg available K kg^−1^, and a pH of 7.24. The soils were sieved with 2-mm mesh and preincubated for 2 weeks (Trabue et al., 2006). The concentration transfer of this study was based on soil depth of 10 cm with a bulk density of 1.5 g cm^−3^ (GB/T31270.1-2014, 2014). The purity of clomazone is 98.4% and purchased from Beijing Qinchengyixin Technology Development Co., Ltd. (Beijing, China). Three clomazone treatments were prepared in brown bottles: 0.8 mg kg^−1^ (active ingredients per soil dry weight; L), 8 mg kg^−1^ (a.i./dw; M), and 80 mg kg^−1^ (a.i./dw; H). The L level represents recommended application rate in the field; the M level represents excessive use of clomazone in the field, and the H level represents extremely polluted soil (e.g., soil near a pesticide factory). In addition, a control treatment was also needed. These treatments were all prepared in triplicate. Soil moisture was adjusted daily by deionized water to 50% of the maximum water-holding capacity. The soil samples were kept for 90 days in an artificial climate box, and the temperature was maintained at 25°C. The samples were taken on days 7, 15, 30, 60, and 90 and kept in a refrigerator at -80°C.

### 2.2. 16s rRNA amplicon sequencing

Soil microbial DNA was extracted using a PowerSoil Isolation Kit (Mo Bio Laboratories, Carlsbad, CA, USA), according to the instructions, and the DNA quality was evaluated using an ND-1000 spectrophotometer (NanoDrop Technologies). 16S rRNA gene was amplified by the primer sets of 341F (5′-CCTAYGGGRBGCASCAG-3′) and 806R (5′-GGACTACNNGGGTATCTAAT-3′) (Yu et al., 2005). Microbial DNA was amplified in 50 μl reactions per sample, and each PCR solution contained 100–300 ng of DNA template, 1.5 μl of each 10 μM primer, 5 μl of 2 mM dNTPs, 1 μl of KOD-Plus-Neo enzyme (Toyobo, Shanghai, China), 5 μl of 10 × PCR Buffer for KOD-Plus-Neo, 3 μl of 25 mM MgSO_4_, and water to 50 μl. Reaction procedures were as follows: an initial step was 94°C and kept for 2 min, followed by 35 cycles of 98°C for 10 s, 62°C for 30 s, and 68°C for 30 s, and the final extension temperature was 68°C for 10 min. Negative-control reactions were also needed. PCR products were analyzed by 1.5% agarose gel electrophoresis and purified with a PCR Purification Kit from QIAGEN (Hilden, Germany). Purified PCR products were sequenced using Illumina equipment (Santiago, CA, USA). Amplicon sequencing data were processed through the USEARCH pipeline (Edgar, 2010, 2013), and clean data were clustered into operational taxonomic units (OTUs) with 97% similarity.

### 2.3. Co-occurrence network construction and characterization

All networks were established on the basis of Pearson's correlations and performed on Cytoscape (Faust and Raes, 2016). The correlation coefficient was set as 0.9. The network topological indices were also based on Cytoscape. For each network, the topological roles of each node were classified and calculated by nodes' within-module connectivity (Zi) and among-module connectivity (Pi) (Guimerà and Nunes Amaral, 2005). An adopted criterion in previous studies (Olesen et al., 2007; Zhou et al., 2011; Shi et al., 2016; Yuan et al., 2021) was used in this study to identify module hubs (Zi ≥ 2.5, Pi < 0.62), connectors (Zi < 2.5, Pi ≥ 0.62), and network hubs (Zi ≥ 2.5, Pi ≥ 0.62). Module hubs referred to nodes that were highly connected to other members in a module, connectors referred to nodes that linked different modules, and network hubs were nodes that were both a module hub and a connector. These nodes were referred to as keystone nodes. Other nodes were classified as peripherals (Banerjee et al., 2019; Röttjers and Faust, 2019).

### 2.4. Network comparison

In a network, the node composition in one network module differs from that in other modules. However, some modules may preserve with some shared nodes after environmental change (Deng et al., 2012). Fisher's exact test is used to evaluate an association of two categorical variables (Warner, 2013) and has been used to identify the preserved modules in previous studies (Horvath, 2011; Langfelder et al., 2011; Deng et al., 2012; Dong et al., 2021). This method has also been used to evaluate preserved modules between control and clomazone treatments in two soils. There were four categories for the nodes within the two networks during the evaluation of preserved modules. In the first case, members were included in two modules; in the second case, members were included in one module of the pair; in the third case, members were included in the other module of the pair; in the fourth case, members were not included in these two modules. To determine whether nodes in the two modules were independent or exclusive, the observed frequency of the four categories was placed into four cells of a contingency table for one-sided exact testing. Each *p*-value from the exact tests was adjusted through the Bonferroni procedure within each network. Through Fisher's exact test, two modules from different networks that both included a significant part of the same nodes were considered as preserved modules.

### 2.5. Network stability

Network stability could evaluate ecological system stability to disturbance (Thébault and Fontaine, 2010), and it was usually evaluated by network robustness and vulnerability (Wu et al., 2021; Yuan et al., 2021). Robustness and vulnerability were used to evaluate network stability (Wu et al., 2021; Yuan et al., 2021). Robustness is defined as the remaining proportion of species after random removal in the network (Montesinos-Navarro et al., 2017). In this study, every 0.05% of nodes was randomly removed to simulate random species removal. Vulnerability is calculated as V = max [(E – Ei)/E], in which E is the global efficiency and Ei is the global efficiency after removing node i and its entire links (Deng et al., 2012). Global efficiency is calculated as E = ∑_j≠*i*_[1/d(i,j)]/n(n – 1), in which d(i,j) is the number of edges in the shortest path of node i to j (Deng et al., 2012).

### 2.6. Picutis functions

The metabolic function of each sample was predicted by tax4fun based on 16S rRNA gene data (Aßhauer et al., 2015). There was one cellular process, three genetic information processing, and 11 metabolism categories that have been used to analyze the correlation with the bacterial community using the Mantel test (Duan et al., 2020). The cellular process was cell growth and death (CGD); the genetic information processing categories were folding, sorting, and degradation (FSD), replication and repair (RR), and translation; and the metabolism categories were carbohydrate metabolism (CM), lipid metabolism (LM), amino acid metabolism (AAM), metabolism of cofactors and vitamins (MCV), xenobiotic biodegradation and metabolism (XBM), biosynthesis of other secondary metabolites (BOSM), energy metabolism (EM), metabolism of terpenoids and polyketides (MTP), metabolism of other amino acids (MOAA), nucleotide metabolism (NM), and glycan biosynthesis and metabolism (GBM). Some functions were fundamental for ecological balance, for example, XBM is important for chemical pollution cleaning (Thelusmond et al., 2019).

### 2.7. Data analysis for network difference

Analysis of similarity (ANOSIM) has been used to evaluate differences in bacterial community structure based on Bray–Curtis distance (Oksanen et al., 2012). Network dissimilarity is an effective tool to evaluate networks' differences, and it is based on network nodes and edges (Poisot et al., 2012; Mo et al., 2021). Shared nodes and edges of two networks are used to evaluate coexisting elements of different networks. Correlation coefficient “r” has been used to evaluate the correlation of functions to the bacterial community; Fisher's least significant difference test has been used to evaluate significant differences, and a 5% level was set (*p* < 0.05).

## 3. Results

### 3.1. Network topological indices

**Figure 1:**
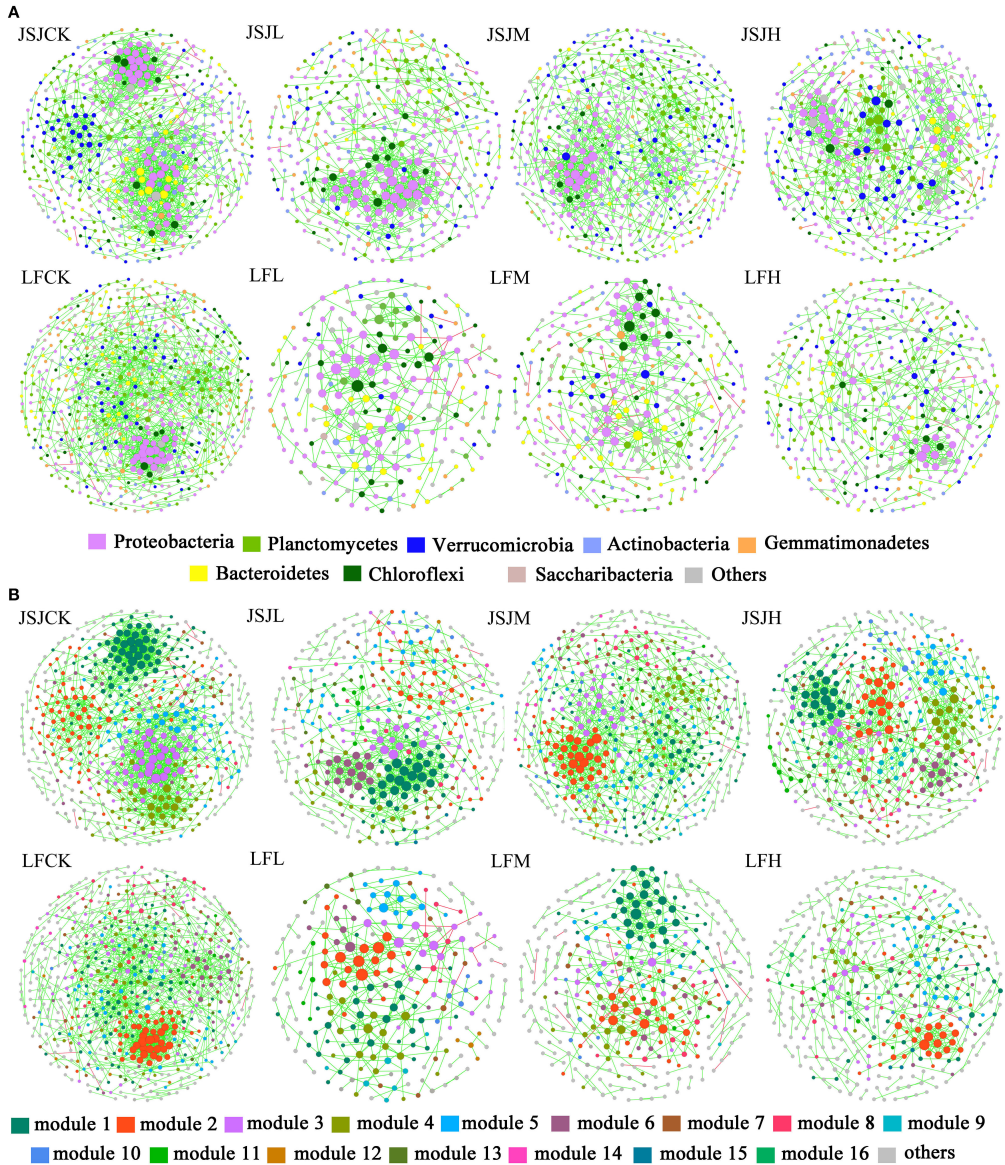
Visualization of bacterial networks for each treatment in the two soils. **(A)** The color of the nodes represents different phyla. **(B)** The color of the nodes represents different modules.

There were eight networks that have been established in Figure 1A. In all networks, the node comprised mostly of eight phyla: Acidobacteria, Actinobacteria, Bacteroidetes, Chloroflexi, Gemmatimonadetes, Planctomycetes, Proteobacteria, and Verrucomicrobia. In the JSJ soil, the node percentages in clomazone treatments were increased in Acidobacteria, Proteobacteria (except for H treatment), and Verrucomicrobia (except for L treatment) but decreased in Actinobacteria, Gemmatimonadetes, Bacteroidetes (except for L treatment), Chloroflexi (except for M treatment), and Planctomycetes (except for M treatment) (Table 1). In the LF soil, Acidobacteria (except for H treatment), Chloroflexi (except for L treatment), Planctomycetes, and Verrucomicrobia were increased, while others were decreased (except for Actinobacteria in H treatment and Proteobacteria in M treatment) (Table 1).

**Table 1:** Percentages of the predominant phyla in CK (control), L (0.8 mg kg^−1^), M (8 mg kg^−1^), and H (80 mg kg^−1^) treatments in the JSJ and LF soils.

	**JSJ**				**LF**			
	**CK**	**L**	**M**	**H**	**CK**	**L**	**M**	**H**
Acidobacteria	3.65%	6.62%	3.91%	6.28%	6.75%	14.65%	7.67%	5.60%
Actinobacteria	11.73%	10.69%	10.18%	10%	8.44%	7.25%	5.01%	8.91%
Bacteroidetes	4.81%	5.85%	4.11%	4.42%	10.97%	4.99%	8.85%	6.87%
Chloroflexi	2.88%	2.04%	2.94%	2.33%	2.53%	2.90%	5.01%	4.83%
Gemmatimonadetes	7.69%	5.09%	4.70%	6.05%	10.97%	7.09%	8.55%	7.89%
Planctomycetes	16.92%	16.03%	19.18%	16.51%	10.55%	22.38%	11.50%	16.03%
Proteobacteria	37.69%	43.26%	38.94%	35.35%	35.86%	26.41%	37.76%	31.04%
Verrucomicrobia	10.96%	7.12%	11.94%	13.72%	4.22%	7.41%	5.60%	8.91%
Others	3.67%	3.30%	4.10%	5.34%	9.71%	6.92%	10.05%	9.92%

The network's topological indices are shown in Table 2. Compared with the network of the JSJ control soil, network size (total nodes), the number of links, and the degree of clomazone treatments were decreased by 9–127, 648–829, and 2.249–2.429, respectively. While in the LF soil, network size (total nodes), the number of links, and the degree of clomazone treatments were decreased by 228–384, 667–753, and 0.703–1.371, respectively.

**Table 2:** Network indices of each network.

	**JSJCK**	**JSJL**	**JSJM**	**JSJH**	**LFCK**	**LFL**	**LFM**	**LFH**
Total nodes	520	393	511	430	621	237	339	393
Total links	1,585	756	937	822	1,083	330	413	416
Average degree	6.096	3.847	3.667	3.823	3.488	2.785	2.437	2.117
Average path distance	13	24	18	13	17	16	15	21

**Figure 2:**
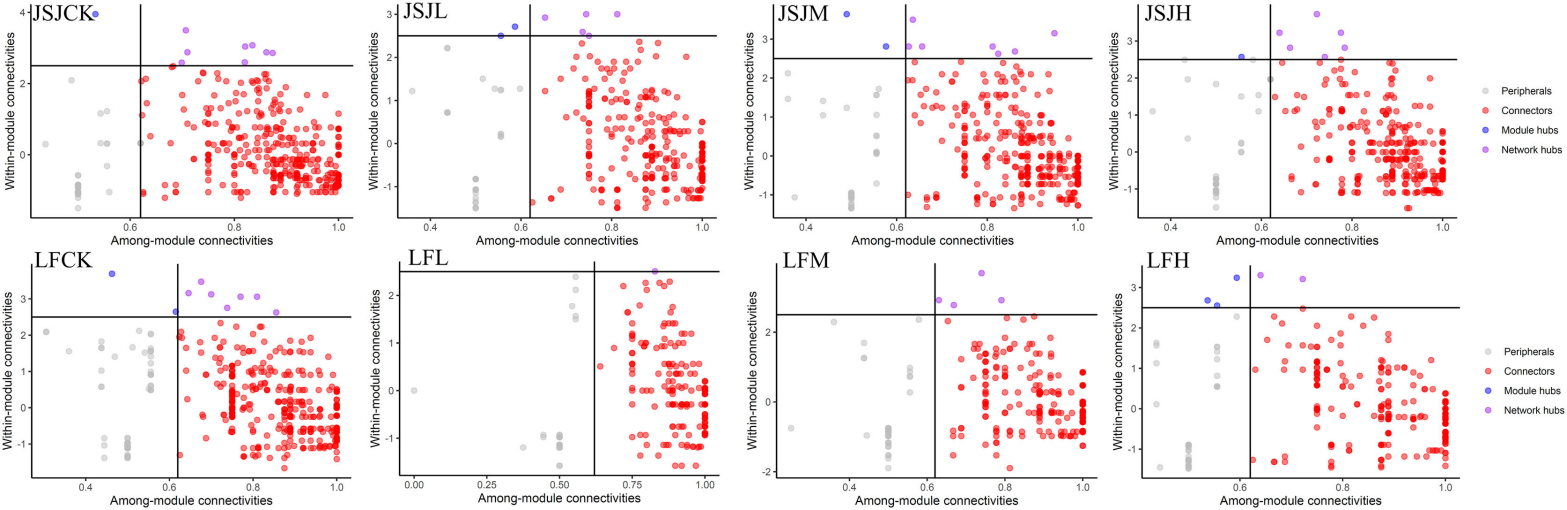
Keystone nodes in different bacterial networks.

**Figure 3:**
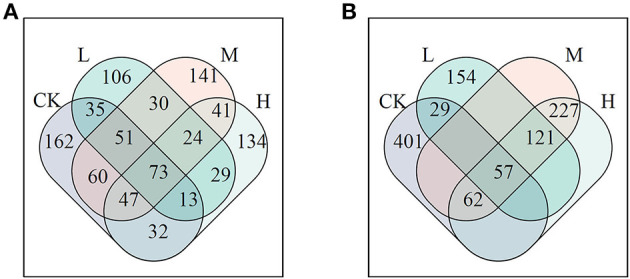
Venn diagram of keystone nodes in each network in the JSJ **(A)** and LF **(B)** soils.

The influenced network could result in changing the roles of the networked members. Based on the criteria of node classification, keystone nodes were 473, 360, 466, and 392 for control, L, M, and H in the JSJ soil, respectively; network keystone nodes were 549, 360, 466, and 466 for control, L, M, and H in the LF soil, respectively (Figure 2). The shared keystone nodes were 173, 231, and 165 for the comparison of control and L, control and M, and control and H in the JSJ soil. The shared keystone nodes were 86, 119, and 119 for the comparison of control and L, control and M, and control and H in the LF soil (Figure 3).

### 3.2. Network dissimilarity, shared nodes, and links

ANOSIM is for the comparison of control and clomazone treatments. They were 0.617–0.879 in the JSJ soil and 0.985–0.997 in the LF soil (Table 3). The network dissimilarities between control and clomazone treatments were 0.954–0.983 in the JSJ soil and 0.979–0.990 in the LF soil (Table 3). These results revealed that the composition of each network was significantly impacted by clomazone in both soils. In addition, the shared nodes between control and clomazone treatments were used to evaluate the effects of clomazone in the JSJ soil and LF soil. Compared with the nodes in the JSJ soil control, the shared nodes were 196, 260, and 194 for L, M, and H treatments, respectively (Table 3). For the LF soil, there were 131, 151, and 171 shared nodes for L, M, and H treatments, compared with the nodes in the control treatments (Table 3). Compared with the links in the JSJ soil control, the shared links were 71, 100, and 36 for L, M, and H treatments, respectively (Table 3). For the LF soil, there were 17, 25, and 12 shared nodes for L, M, and H treatments, compared with the nodes in the control treatments (Table 3).

**Table 3:** ANOSIM, network dissimilarity, shared nodes, and links of the networked communities between control and clomazone treatments.

		**ANOSIM**	**Network dissimilarity**	**Shared nodes**	**Shared links**
JSJ	CK vs. L	0.708	0.964	196	71
	CK vs. M	0.617	0.954	260	100
	CK vs. H	0.879	0.983	194	36
LF	CK vs. L	0.997	0.985	131	17
	CK vs. M	0.997	0.979	151	25
	CK vs. H	0.985	0.990	171	12

### 3.3. Network stability

**Figure 4:**
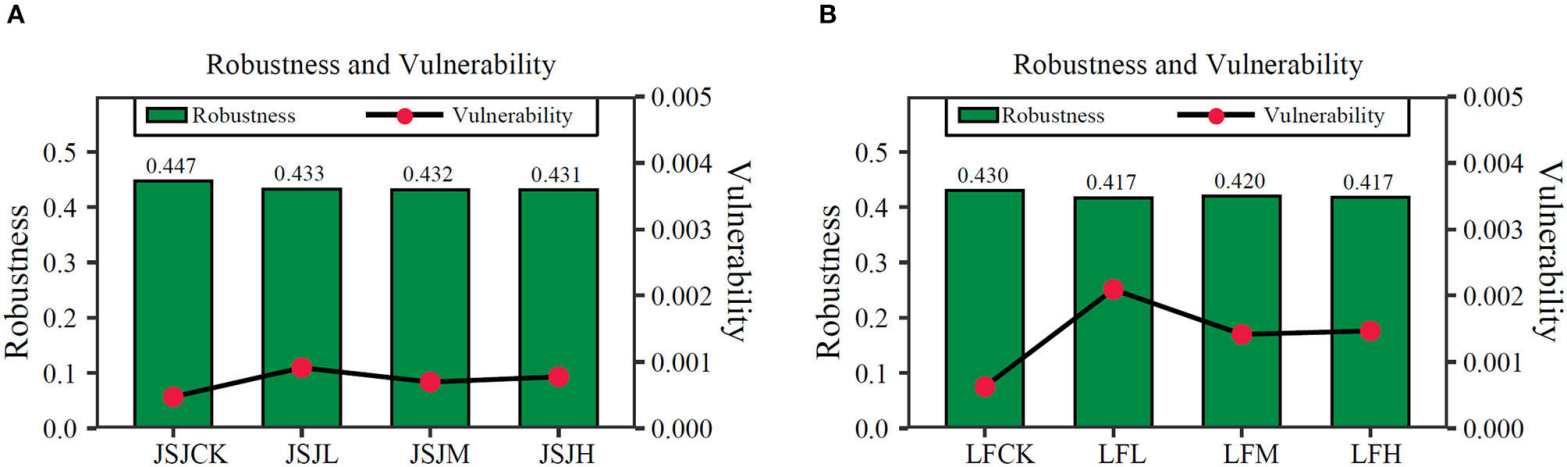
Robustness and vulnerability of networks in the JSJ **(A)** and LF **(B)** soils.

Based on random species loss, the robustness was decreased by 0.014–0.016 in clomazone treatments in the JSJ soil; for the LF soil, it was decreased by 0.01–0.013 in clomazone treatments (Figure 4). For vulnerability in the JSJ soil, it was increased by 0.00079–0.00147 in clomazone treatments; in the LF soil, it was decreased by 0.00023–0.00044 for clomazone treatments.

### 3.4. Network organization

The influenced networks suggested that clomazone could alter network organization. The big modules (i.e., ≥5 nodes) were used to analyze preserved modules based on Fisher's exact test. In total, there were 25 preserved module pairs in the two soils (Table 4), and most of the preserved module individuals belong to the phyla of Proteobacteria, Acidobacteria, and Planctomycetes (Figures 1A, B). Specifically, in the JSJ soil, there were five module pairs accounted for 0.39% of total module pairs between CK and L, seven module pairs accounted for 0.55% of total module pairs between CK and M, and six module pairs accounted for 0.58% of total module pairs between CK and H (Table 4). In the LF soil, there were two module pairs accounted for 0.13% of total module pairs between CK and L, three module pairs accounted for 0.18% of total module pairs between CK and M, and two module pairs accounted for 0.09% of total module pairs between CK and H (Table 4).

**Table 4:** Preserved module pairs of the JSJ soil.

**Preserved module pairs**	**Overlapping nodes**	**Nodes only in module 1**	**Nodes only in module 2**	**Nodes absent from both modules**
JSJCK_M1:JSJL_M3	13	67	23	614
JSJCK_M3:JSJL_M2	13	54	29	621
JSJCK_M1:JSJL_M1	27	53	16	621
JSJCK_M1:JSJL_M6	11	69	11	626
JSJCK_M7:JSJL_M11	5	9	2	701
JSJCK_M3:JSJM_M1	17	50	41	663
JSJCK_M1:JSJM_M2	36	44	21	670
JSJCK_M7:JSJM_M3	6	8	41	716
JSJCK_M2:JSJM_M4	13	63	31	664
JSJCK_M6:JSJM_M4	9	8	35	719
JSJCK_M3:JSJM_M5	12	55	29	675
JSJCK_M2:JSJM_M6	15	61	20	675
JSJCK_M1:JSJH_M3	16	64	26	650
JSJCK_M1:JSJH_M1	16	64	40	636
JSJCK_M3:JSJH_M4	14	53	22	667
JSJCK_M7:JSJH_M9	6	8	1	741
JSJCK_M5:JSJH_M8	8	50	10	688
JSJCK_M2:JSJH_M13	4	72	1	679
LFCK_M3:LFL_M4	7	42	13	665
LFCK_M2:LFL_M2	11	47	13	656
LFCK_M1:LFM_M2	11	60	26	712
LFCK_M2:LFM_M1	23	35	23	728
LFCK_M1:LFM_M4	10	61	9	729
LFCK_M2:LFH_M2	13	45	18	767
LFCK_M6:LFH_M5	6	35	12	790

CK, control; L (0.8 mg kg^−1^), M (8 mg kg^−1^), and H (80 mg kg^−1^) clomazone used for experimentation. M before figures represents network module.

### 3.5. Connection of bacterial communities to functions

**Figure 5:**
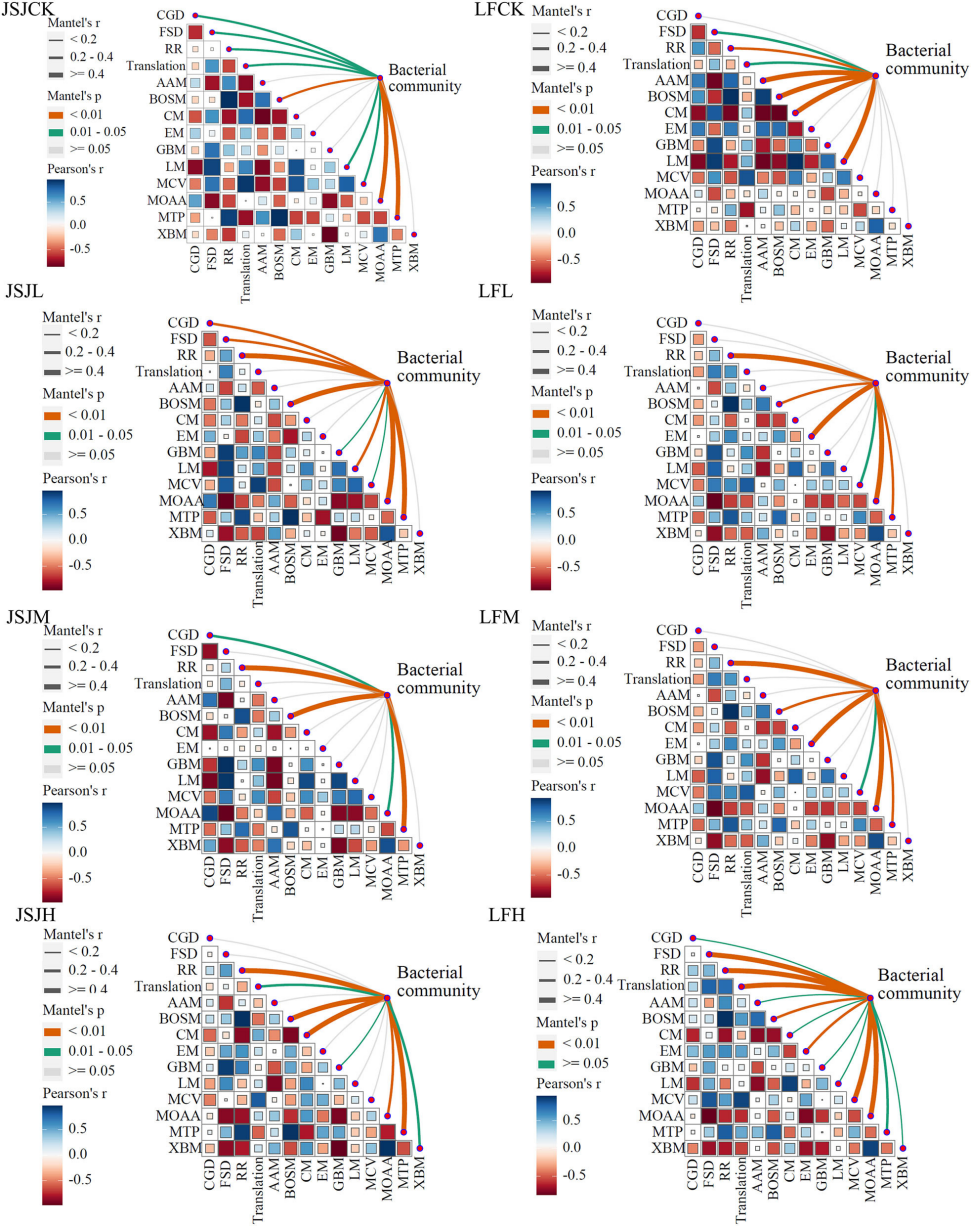
Relationships between bacterial network communities and functions for each treatment in the two soils.

An intriguing issue is whether the alterations in bacterial network composition as a result of clomazone treatment caused alterations in microbial community functions and their associated ecosystem processes. We used the Mantel test to address this issue, and the relationships are shown in Figure 5. In the JSJ soil, bacterial network community correlated with MOAA and MTP in the control treatment (*r* ≥ 0.4); in clomazone treatments, the correlations of MOAA (only in L treatment), BOSM, RR, MTP, and CM (only in H treatment) with the bacterial community are higher than 0.4. In the LF soil, bacterial network community correlated with BOSM, CM, AAM, and LM in the control treatment (*r* ≥ 0.4); in L treatment, it correlated with EM, RR, and MOAA; in M treatment, it correlated with EM; in H treatment, it correlated with translation, RR, MCV, MOAA, and FSD (*r* ≥ 0.4).

## 4. Discussion

Soil microbiome is an ecological system that is important in material cycling and nutrient maintaining. In an ecological system, there are complicated relationships. The microbial network has gradually been used to evaluate these complicated relationships (Ze et al., 2013; Przulj and Malod-Dognin, 2016; Mo et al., 2021). In this study, the influenced network complexities suggested that clomazone significantly altered bacterial network composition and relationships. The amount of total nodes in clomazone treatment networks indicated that the disconnected bacterial species were increased by clomazone. The significantly decreased links and average degrees also indicated that the connections of the network species were decreased. The most possible reason, due to some bacterial species, could use clomazone as a carbon resource and increase their abundance, and some bacterial species have been inhibited by clomazone or other bacterial species. Different effects of bacterial species' abundance were the reasons for the impacted bacterial connections. Therefore, that is the reason of impacted bacterial network topological indices, stability, and organization. In the study by Zhang et al. (2021), thiamethoxam also decreased bacterial network nodes, links, and average degrees. These influences by clomazone also induced the topological role of the network nodes to change.

The impacted network indices suggested that bacterial network composition has been changed. The results of ANOSIM, network dissimilarity, and shared nodes confirmed this inference. ANOSIM has been used to evaluate the difference between different network communities (Yuan et al., 2021). In the study by Yuan, they used ANOSIM to analyze whether the network composition was changed by climate warming (Yuan et al., 2021). Network dissimilarity has first been published by Poisot et al. (2012) and has also been used by other researchers to evaluate network dissimilarity (Mo et al., 2021; Liao et al., 2023). For example, Liao et al. (2023) analyzed the difference in marine medaka gut and gill microbial networks by network dissimilarity; in the study by Mo et al. (2021), they used shared node and edges and network dissimilarity to evaluate the difference in microeukaryotic plankton network in different salinity in the subtropical urban reservoir. These results suggested that ANOSIM and network dissimilarity are effective in evaluating bacterial network composition dissimilarity.

The impacted network indices and composition suggested that the bacterial network stability of the soils has been impacted. Network robustness and vulnerability were always used to evaluate network stability (Wu et al., 2021). In this study, the decreased network robustness and increased vulnerability suggested that bacterial network stability was decreased by clomazone. It also suggested that the resistance of the bacterial network to disturbance was decreased, and more species will lose from the connected network in clomazone-treated soils. The connection and cooperation of bacterial species will be more fragile after clomazone treatment. The decreased edges of networks of all clomazone treatments suggested that decreased edges should be responsible for decreased network stability (Yuan et al., 2021). Microbial network stability is important in ecosystem function (Coyte et al., 2015; Pan et al., 2023). The profile of network modules and functions proved this suggestion.

Normally, most species in the network will cluster as modules and the species exert their functions through modules (Segal et al., 2003; Banerjee et al., 2018). This demonstrates that preserved network modules will preserve some functions (Yuan et al., 2021). There were more shared nodes and links between control and clomazone treatments in the JSJ soil which suggested that more modules will preserve in the JSJ soil. This suggestion has been proven in the results of the preserved modules in the two soils. However, there were fewer modules preserved after clomazone treatment. These suggested that the functions of bacterial network have been changed. The relationships between network community and functions further proved this indication. In both soils, the correlation of network community with functions suggested that the functions of the bacterial community have been changed. In the JSJ soil, the function diversity of the bacterial network community was increased by clomazone with more functions correlated with the network community. In addition, MTP was significantly correlated with the bacterial community in all treatments in the JSJ soil which suggested that the bacterial network community function of MTP was stable in facing clomazone. Soil bacterial functions are sensitive to pesticides and have been improved by another study (Han et al., 2022). In the study by Han et al. (2022), boscalid significantly impacted N cycling genes.

## 5. Conclusion

In this study, we used network complexities, composition, keystone node, and stability to analyze the impact of clomazone on soil bacterial networks. The results indicated that clomazone decreased bacterial network nodes, links, and average degrees. Clomazone impacted the bacterial network composition, and the topological role of the nodes was also impacted according to keystone nodes. The decreased robustness and increased vulnerability suggested that network stability was increased by clomazone. Preserved modules and the correlation of bacterial network community to soil bacterial functions manifest that the functions of the bacterial network community have been changed. Overall, the soil bacterial network has been significantly changed by clomazone.

## Data availability statement

The datasets presented in this study can be found in online repositories. The names of the repository/repositories and accession number(s) can be found below: NCBI - SAMN08721528-SAMN08721647.

## Author contributions

HH and PD conceived and wrote this manuscript. HH, PD, and JH performed the bioinformatics analyses. ZZ, XZ, and WF revised this manuscript. All authors contributed to the article and approved the submitted version.

## Footnotes

^1^ http://sitem.herts.ac.uk/aeru/ppdb/en/Reports/168.htm
